# Prenatal diagnosis of recurrent hypoplastic left heart syndrome associated with *MYH6* variants: a case report

**DOI:** 10.1186/s12872-023-03169-z

**Published:** 2023-03-08

**Authors:** B. Najib, T. Quibel, A. Tessier, J. Mortreux, P. Bouvagnet, C. Cohen, F. Vialard, R. Dard

**Affiliations:** 1 Department of Obstetrics and Gynecology, Saint Joseph University, Beirut, 0000 Lebanon; 2 Department of Obstetrics and Antenatal Fetal Medicine, Centre Hospitalier Intercommunal de Poissy-Saint-Germain-en-Laye, 78300 Poissy, France; 3 Department of Genetics, Centre Hospitalier Intercommunal de Poissy-Saint-Germain-en-Laye, 78300 Poissy, France; 4 Service de Génétique, Laboratoire Eurofins Biomnis, 69007 Lyon, France; 5 RHuMA, UMR-BREED, INRA-ENVA-UVSQ, 78180 Montigny Le Bretonneux, France

**Keywords:** Hypoplastic left heart syndrome, *MYH6*, *MYH7*, Congenital heart disease, Case report

## Abstract

**Background:**

Hypoplastic left heart syndrome (HLHS) is a rare but genetically complex and clinically and anatomically severe form of congenital heart disease (CHD).

**Case presentation:**

Here, we report on the use of rapid prenatal whole-exome sequencing for the prenatal diagnosis of a severe case of neonatal recurrent HLHS caused by heterozygous compound variants in the *MYH6* gene inherited from the (healthy) parents. *MYH6* is known to be highly polymorphic; a large number of rare and common variants have variable effects on protein levels. We postulated that two hypomorphic variants led to severe CHD when associated in trans; this was consistent with the autosomal recessive pattern of inheritance. In the literature, dominant transmission of *MYH6*-related CHD is more frequent and is probably linked to synergistic heterozygosity or the specific combination of a single, pathogenic variant with common *MYH6* variants.

**Conclusions:**

The present report illustrates the major contribution of whole-exome sequencing (WES) in the characterization of an unusually recurrent fetal disorder and considered the role of WES in the prenatal diagnosis of disorders that do not usually have a genetic etiology.

## Background

The first-trimester prenatal diagnosis of congenital heart disease (CHD) using transvaginal ultrasound was first reported in 1990s [1]. Since then, various groups with a high risk of CHD have been defined: these include increased nuchal translucency in the fetus, certain maternal illnesses (e.g., diabetes mellitus), conception via assisted reproductive technology, exposure to teratogenic drugs, an unambiguous family history and/or previous pregnancies with CHD [2]. In the fetal echocardiogram, the ventricles should be symmetric and equivalent in volume; any differences in size are suggestive of disease [3]. However, first-trimester fetal cardiac ultrasound has a limited ability to diagnosing subtle, minor, late-onset or progressive fetal pathologies such as ventricular septal defects, cardiac tumors, valvular stenosis or regurgitation, and coarctation of the aorta [4].

Hypoplastic left heart syndrome (HLHS) is a clinically and anatomically severe form of CHD [5]. It accounts for around 4% of cases of CHD but is responsible for 15–25% of the associated deaths [6]. HLHS combines atresia or stenosis of the aortic or mitral valves with hypoplasia of the left ventricle and the ascending aorta [7].

Although the cause of HLHS is unknown, severe cardiomyopathy is thought to be a complex genetic disorder [7]; the genetic analysis of families with inherited left heart abnormalities (including HLHS, a bicuspid aortic valve, and coarctation of the aorta) have identified a few genes in which mutations are likely to be causative (notably *NKX2.5* [8], *NOTCH1* [9–11] and *MYH6* [5, 12, 13]).

The present report highlights the use of rapid prenatal whole-exome sequencing (WES) to diagnose a severe case of recurrent, neonatal HLHS (cause by compound heterozygous *MYH6* variants) early in pregnancy. Written informed consent was obtained from the patient for publication of this case report and any accompanying images.

## Case presentation

**Fig. 1:**
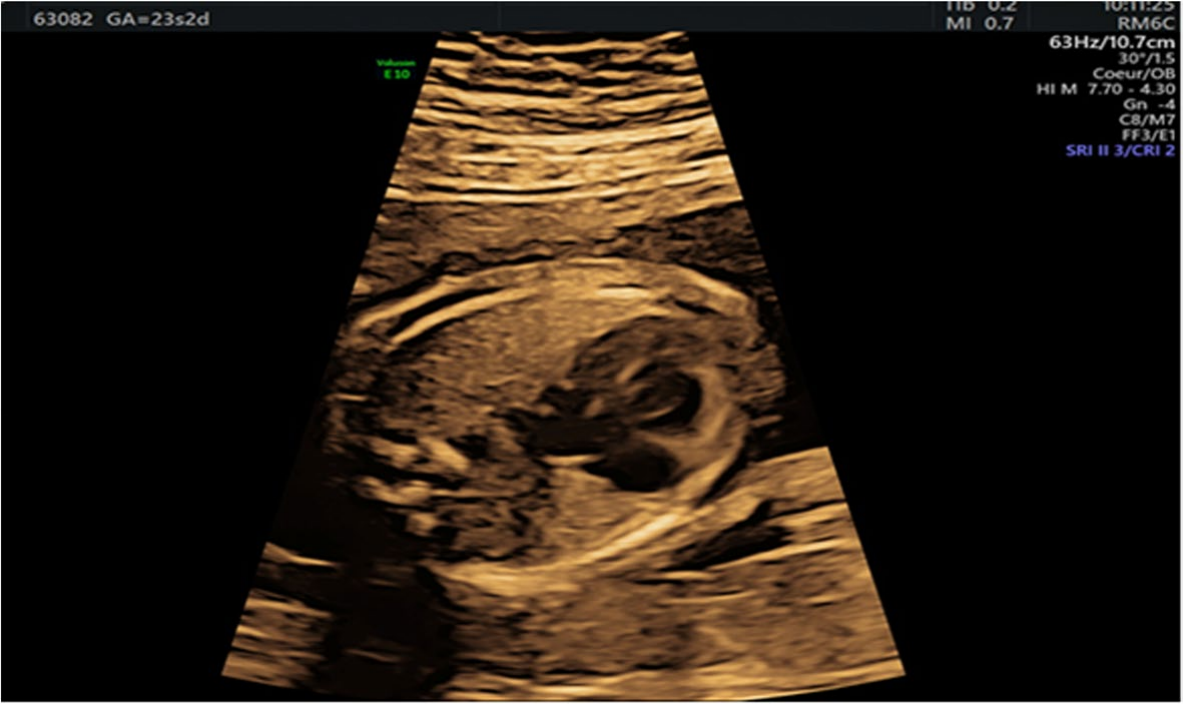
Severe left hypoplastic heart syndrome

We report on the case of a 40-year-old, nonsmoking, nulliparous, Caucasian woman (BMI: 31 kg/m^2^) with an unremarkable medical history other than idiopathic hypertension (treated with labetalol 200 mg once daily). After spontaneous conception, the woman was referred to our center in April 2020 for an undefined fetal cardiopathy. The results of the first-trimester ultrasound scan were normal (crown rump length: 63.68 mm; nuchal translucency: 1.7 mm), with a low risk of trisomy 21 (1 in 1548; PAPP-A: 0.65 multiples of the median, β-HCG: 0.59 multiples of the median) and a negative, non-invasive prenatal test requested by the woman. The diagnosis of severe HLHS with mitral and aortic stenosis was made at 23 weeks of gestation (w.g.) (Fig. 1). The results of a chromosomal microarray (CMA) analysis of an amniotic fluid sample were normal. The couple requested termination of pregnancy at 25 w.g.; given the severe prognosis, the request was approved by the multidisciplinary antenatal board. The couple did not allow a fetal autopsy to be performed.

The parents had normal cardiac ultrasound findings and did not report a family history of CHD or adult cardiomyopathy. The parents were reassured by the initial genetic counselling because HLHS is usually sporadic; in the absence of a family history, the risk of recurrence is 2–4% [14].

**Fig. 2:**
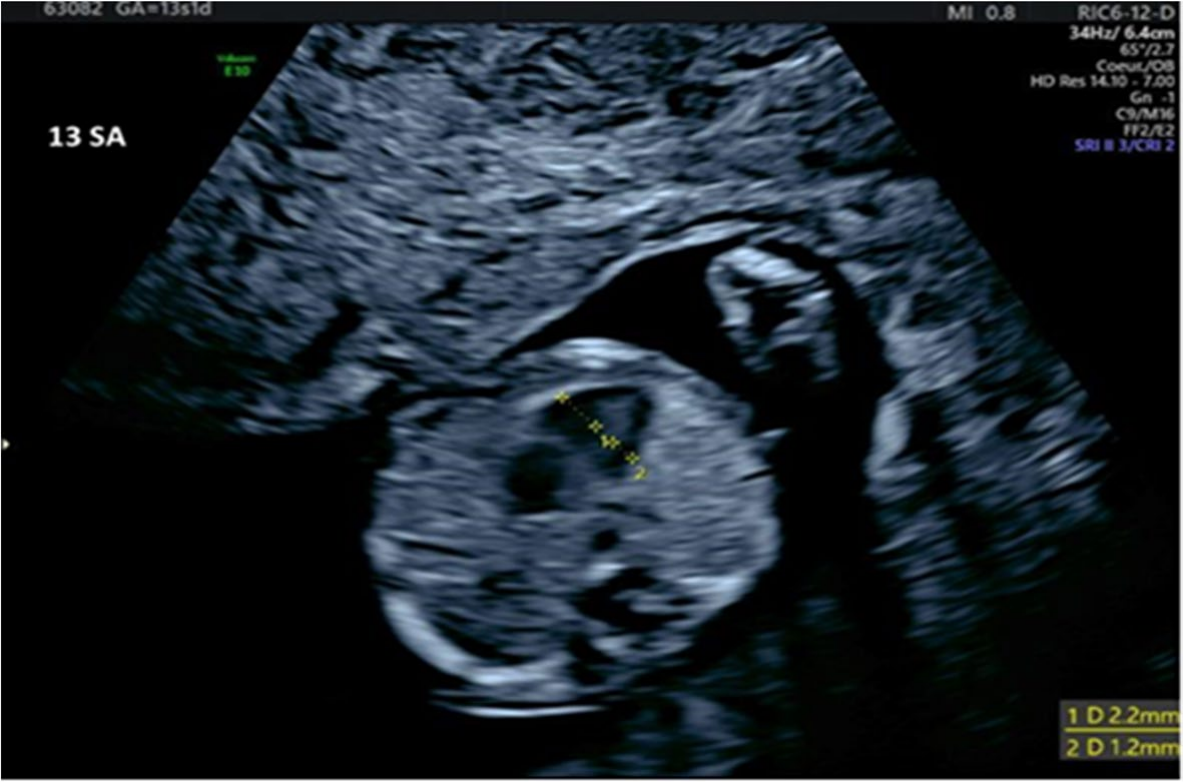
Cardiac asymmetry

**Fig. 3:**
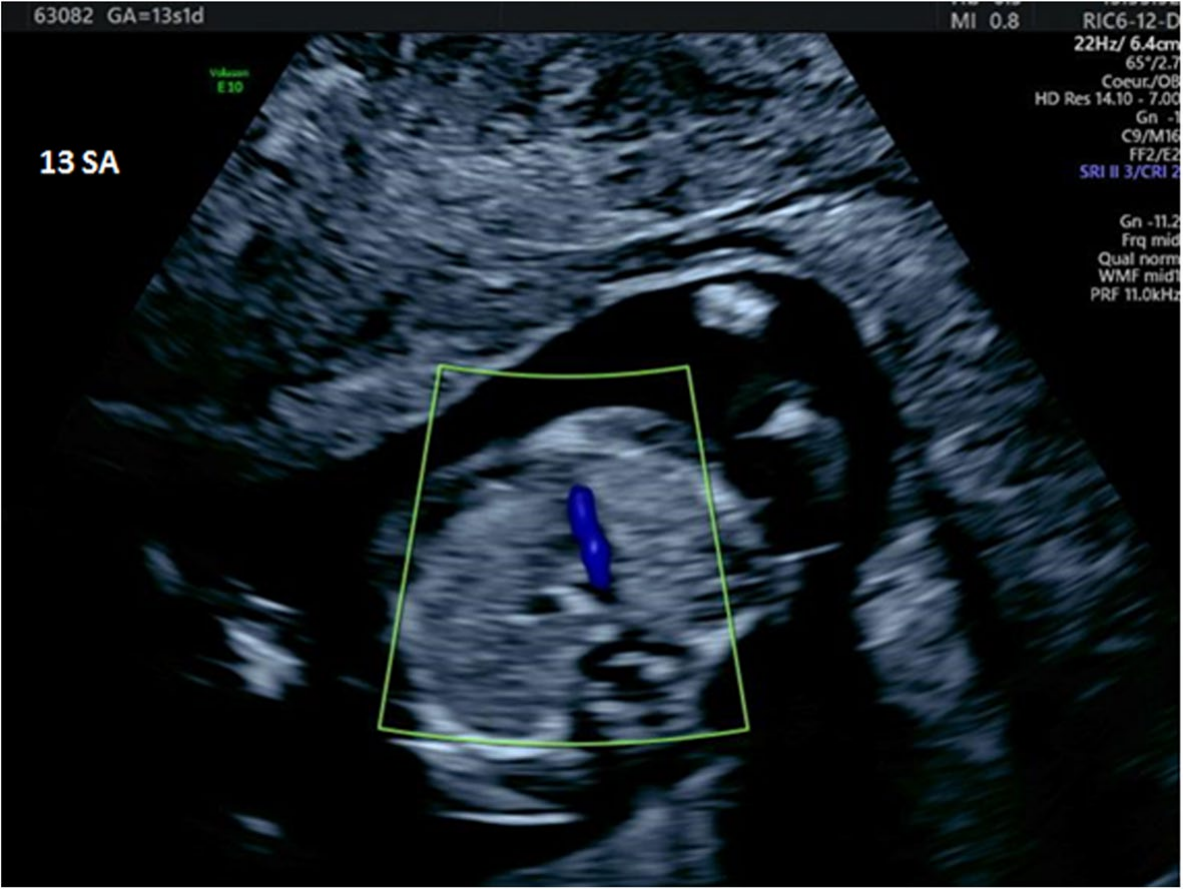
Severe aortic stenosis

A year later (in April 2021, after 13 w.g.), the women was referred for an early morphological ultrasound check-up on her next pregnancy, which revealed cardiac asymmetry and aortic stenosis (Figs. 2 and 3). The diagnosis of HLHS was confirmed at 15 w.g. Again, the results of a CMA analysis of an amniotic fluid sample were normal.

In view of the recurrence of HLHS, we suggested WES. The couple agreed, and a quartet analysis was performed on fetal DNA stored from the previous pregnancy, DNA from the ongoing pregnancy, and the parents’ DNA. WES revealed that both fetuses were compound heterozygous for probably pathogenic *MYH6* variants (class 4, according to the American College of Medical Genetics and Genomics (ACMG) classification [15]) inherited from the mother and the father. The mother carried *MYH6* (NM_002471.4) chr14: g.23853649A > T c.5565 + 2 T > A, predicted to be a loss-of-function (splice donor) variant that was not present in population databases [16]. The father carried *MYH6* (NM_002471.4) chr14:g.23857395G > T c.4328C > A p.(Ala1443Asp) – a very rare missense variant that is predicted to be pathogenic, according to the ACMG classification and the Varsome database (https://varsome.com/). Furthermore, the wild-type and mutant amino acids’ respective physicochemical properties are very different (Grantham score: 126, on a 1-to-215 scale), and the alanine at position 1443 is highly conserved from *Tetraodon nigroviridis* to *Homo sapiens.* Before receiving the WES results, the severe prognosis for HLHS prompted the woman to request termination of pregnancy at 16 w.g.

## Discussion & conclusions

Cardiac muscle myosin consist of two heavy chain subunits, two light chain subunits, and two regulatory subunits. *MYH6* codes for myosin heavy chain α (MHC-α), the major myosin isoform in the fetal heart [17]. After birth, MHC expression switches to *MYH7*, located 4 kb downstream of *MYH6* and which codes for MHC-β. Relative to MHC-β, MHC-α has 1.5 to 3 times more contractile velocity and a 0.6-fold shorter actin attachment duration. In the adult heart, MHC-β expression is predominates in the ventricles and MHC-α expression predominates in the atria.

**Fig. 4:**
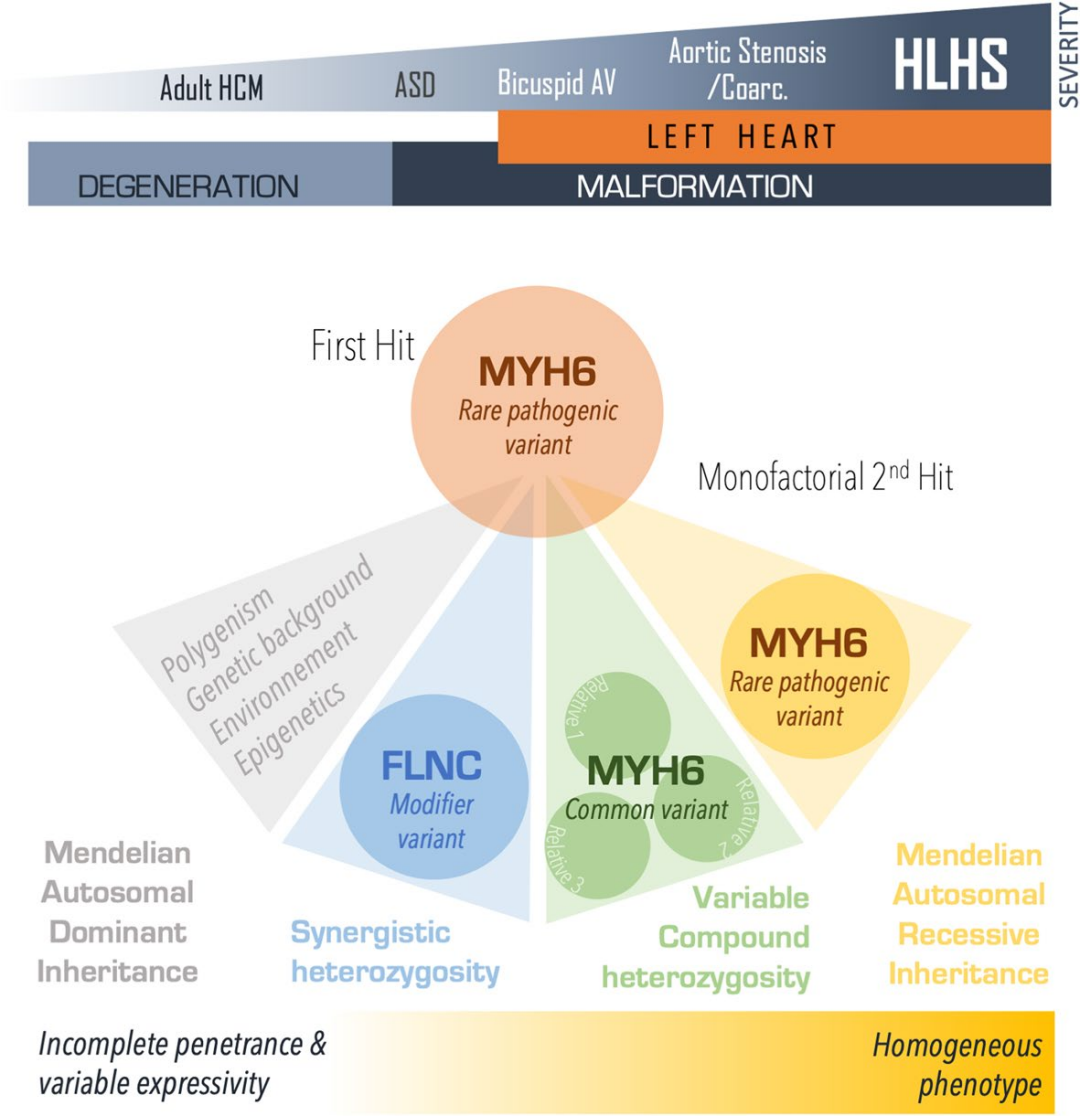
The genotype–phenotype relationship for *MYH6* in the “second variant” hypothesis. mutations in *MYH6* is responsible for degenerative heart diseases or cardiac malformations of varying severity. Three main genotypes are thought to be responsible for these diseases: (i) classical autosomal-recessive inheritance of two rare, pathogenic *MYH6* variants, responsible for the most severe phenotype; (ii) synergistic heterozygosity, i.e. the combination of a single pathogenic *MYH6* variant with a modifier variant in an interacting gene (*FLNC* in this case), and (iii) variable compound heterozygosity, i.e. the combination of a single *MYH6* pathogenic variant with one or more common *MYH6* variants, with intrafamily variability. ASD: atrial septal defect, AV: aortic valve, Coarc: coarctation, FLNC: filamin C, HCM: hypertrophic cardiomyopathy, HLHS: hypoplastic left heart syndrome

Pathogenic *MYH6* variants have been described in several cardiac diseases. For example, it has been shown that heterozygous variants are responsible for moderately severe types of CHD, such as atrial septal defects and AVSD [18] whereas homozygous or compound heterozygous variants are responsible for severe CHD such as HLHS [19]. *MYH6* is also thought to be involved in adult familial hypertrophic cardiomyopathy. The association between *MYH6* variants and HLHS has already been reported; in a study of 190 unrelated HLHS subjects, rare *MYH6* variants were observed in around 10% of cases [5]. More recently, whole-genome sequencing revealed a highly significant (*p* = 0.000068), abnormally high proportion of pathogenic *MYH6* variants observed in 197 patients with HLHS (vs. 813 controls) [13]. The frequency of *MYH6* variants was also 10% in this population of patients with HLHS. Despite the presence of autosomal dominant transmission of left heart disease/cardiomyopathy in some families, a single pathogenic *MYH6* variant alone might not be enough to cause disease: a second hit might be necessary. Interestingly, the second hit might be either a second rare *MYH6* pathogenic variant (i.e. compound heterozygosity), a “common” *MYH6* variant (minor allele frequency > 0.01) or a variant in a modifier gene like *FLNC* (i.e. synergistic heterozygosity). These various possibilities might explain the broad phenotypic spectrum associated with *MYH6* variants (Fig. 4), which ranges from a normal heart to a severe form of CHD like HLHS. As reported for many genes with a high variant density, mutations in *MYH6* and *CFTR* are probably associated with a broad spectrum of effects and marked phenotypic variability.

Discordant inheritance patterns have been reported in the literature. The versatile nature of MYH6-related inheritance prompts us to think that MYH6 is at the interface between Mendelian autosomal-dominant inheritance and autosomal-recessive inheritance and thus has an unconventional pattern of inheritance.

The “common variant hypothesis” has been further underpinned by several recent reports of an association between rare variants and common variants in MYH6-related conditions [20] and for other genes responsible for congenital left heart defects [21]. This hypothesis is also supported by the results of several genome-wide association studies [22–24], in which MYH6 polymorphisms were linked to cardiac disease in the general population; hence, a common variant might have harmful consequences. In the overall genetic landscape and along with environmental factors and modifier genes, the “common variant hypothesis” tends to explain incomplete penetrance and the variable expressivity of Mendelian autosomal-dominant diseases [25].

Cases of compound heterozygosity or homozygosity for *MYH6* mutations are rare; to the best of our knowledge, the only literature report concerned cases in four families [18]. Most of these cases could have been due to synergistic heterozygosity or an association with a common *MYH6* variant. It should be noted that *MYH6* is one of the most variable genes, and so there are probably many common alleles. In the case described here, neither the fetus nor the parents carried a “common” *MYH6* or *FLNC* variant; this is consistent with the absence of a family history of CHD and hypertrophic cardiomyopathy and the classical autosomal recessive transmission of HLHS.

Moreover, the presence of *MYH6* variants is associated with greater expression of MYH7, which results in hypocontractility [5]. According to the literature, CHD is associated with low levels of *MYH6* mRNA and higher levels of *MYH7* mRNA [26].

There are two possible explanations for a disease-causing effect of *MYH6* variants. Firstly, severe valvular stenosis might led to ventricular dysmorphology and reduced preload [7]. Secondly, the defective expansion and/or differentiation of cardiomyocytes might result in ventricular dysmorphology and dysfunction [27]. In the present case, we observed left hypoplastic heart failure with mitral and aortic stenosis; these observations are compatible with the first hypothesis. The presence of *MYH6* variants is strongly associated with *MYH7* overexpression in both atrial and ventricular tissues [5], leading to hypocontractility. Delayed fetal blood flow from the right atrium to the left atrium and through the mitral valve would then result in limited filling of the left ventricle and thus HLHS.

The present report is the first to have described the detection of *MYH6* variants related to fetal HLHS during an ongoing pregnancy. Our case also emphasizes the value of WES for understanding the unusual recurrence of fetal anomalies during pregnancy. Fetal anomalies are detected in 3% of all pregnancies and are responsible for 20% of prenatal deaths [28]. Thus, some cases with an abnormal obstetric history and/or ultrasound anomalies but normal karyotyping and CMA results have been underdiagnosed [29]. WES improves the reliability of prenatal diagnosis and facilitates genetic counseling and medical decisions during early pregnancy [30]. Furthermore, WES improves the diagnostic yield for recurrent fetal structural anomalies and can reveal new genes that are potentially relevant in human development [31]. The present report illustrates the major contribution of WES in the characterization of an unusually recurrent fetal disorder and considered the role of WES in the prenatal diagnosis of disorders that do not usually have a genetic etiology. A better understanding of genetic mechanisms and WES results is crucial for future medical practice.

## Data Availability

Upon request by contact to the corresponding author Rodolphe.dard@gmail.com.

## Abbreviations

HLHS: Hypoplastic left heart syndrome
CHD: Congenital heart disease
WES: Whole-exome sequencing
ACMG: American College of Medical Genetics and Genomics
